# Based a machine learning approach to investigate the factors influencing nirmatrelvir/ritonavir exposure in human plasma: a multicenter, observational study

**DOI:** 10.3389/fcimb.2026.1744619

**Published:** 2026-03-06

**Authors:** Yue Zhang, Xiner Yang, Liang Sun, Runcong Zhang, Weibin Fan, Nengming Lin, Bin Lin

**Affiliations:** 1 College of Pharmaceutical Sciences, Changxing People’s Hospital, Zhejiang Chinese Medical University, Huzhou, Zhejiang, China; 2 Key Laboratory of Intelligent Pharmacy and Individualized Therapy of Huzhou, Huzhou, China; 3 Department of Pharmacy, The Third Changxing People’s Hospital, Huzhou, Zhejiang, China; 4 Key Laboratory of Clinical Cancer Pharmacology and Toxicology Research of Zhejiang Province, Affiliated Hangzhou First People’s Hospital, School of Medicine, Westlake University, Hangzhou, China; 5 Key Laboratory of Multiple Organ Failure, Zhejiang University, Ministry of Education, Hangzhou, China

**Keywords:** COVID -19, machine learning, multivariate prognostic models, nirmatrelvir/ritonavir, therapeutic drug monitoring

## Abstract

**Objectives:**

Nirmatrelvir/ritonavir (N/R) is an effective antiviral for treating COVID-19. However, evidence supporting therapeutic drug monitoring (TDM) for N/R remains limited, potentially increasing the risk of adverse reactions and compromising efficacy. This study aims to identify factors influencing N/R plasma exposure and to develop and internally validate a machine learning model for predicting N/R concentrations, thereby supporting individualized therapy.

**Methods:**

We retrospectively analyzed data from 139 patients who received N/R at two centers. Baseline clinical and laboratory variables were collected, and steady-state trough concentrations of nirmatrelvir and ritonavir were measured on day 3 of treatment. Logistic regression was used to examine the association between drug concentration and prognosis. After excluding highly correlated features, a random forest model identified key factors affecting drug exposure. An XGBoost regression model was then constructed with the selected features, and its predictive performance was evaluated using mean absolute error (MAE), mean squared error (MSE), root mean squared error (RMSE), and R². Five-fold cross−validation was applied for internal validation.

**Results:**

Nirmatrelvir trough concentration was not predictive of patient outcomes (AUC = 0.467). Six factors were consistently identified as important determinants of N/R exposure: estimated glomerular filtration rate (eGFR), creatine kinase (CK), aspartate aminotransferase (AST), alanine aminotransferase (ALT), lymphocyte count (Lymph), and procalcitonin (PCT). Ultimately, the evaluation of the predictive model resulted in a mean absolute error (MAE) of 0.717, mean squared error (MSE) of 1.328, root mean squared error (RMSE) of 1.152, and coefficient of determination (R-squared) of 0.779. The prediction model performs well and can provide risk prediction for medication management for N/R, as well as assist in personalized medication.

**Conclusions:**

We identified a set of variables that affect the treatment of N/R through therapeutic drug monitoring and established a machine learning model capable of predicting N/R concentrations with satisfactory performance. These findings provide a basis for integrating TDM with multivariable prediction tools to personalize N/R dosing and improve medication safety.

## 1 Introduction

COVID-19, caused by severe acute respiratory syndrome coronavirus 2019 (SARS-CoV-19), poses a significant threat to global public health systems. Antiviral drugs, including remdesivir (Elsawah et al., 2021), lopinavir/ritonavir (Amani et al., 2021), molnupiravir (Jayk Bernal et al., 2022), and nirmatrelvir/ritonavir (N/R) (Burki, 2022), have demonstrated promising therapeutic effects against COVID-1. N/R, an oral antiviral drug manufactured by Pfizer in the United States, is considered an ideal treatment for mild to moderate COVID-19 and severe disease risk factors (Amani and Amani, 2023; Najjar-Debbiny et al., 2023). N/R consists of two active substances: nirmatrelvir and ritonavir. Nirmatrelvir is a potent inhibitor of SARS-CoV-2 protease, which hinders the processing of viral polyproteins and prevents viral replication. Ritonavir, on the other hand, primarily inhibits the cytochrome P450 3A4 enzyme (CYP3A4), preventing premature metabolic inactivation of nirmatrelvir and increasing its serum concentrations (Martens-Lobenhoffer et al., 2022). Thus, the combination of nirmatrelvir and ritonavir can enhance the effectiveness of nirmatrelvir in alleviating clinical symptoms in patients with COVID-19.

Recent real-world studies have provided robust evidence supporting the effectiveness of nirmatrelvir/ritonavir in diverse patient populations and clinical settings. In a population-based cohort study conducted in Hong Kong during the Omicron wave, Lui et al. demonstrated that N/R use was associated with a 29% lower risk of all-cause mortality and hospitalization among nonhospitalized patients with type 2 diabetes and SARS-CoV-2 infection, a subgroup historically underrepresented in clinical trials (Lui et al., 2023). Similarly, a nationwide study in Greece by Paraskevis et al. including outpatients aged ≥65 years at high risk for disease progression reported that N/R significantly reduced the risk of hospitalization (odds ratio [OR], 0.31) and COVID-19-associated death (OR, 0.28), with the most pronounced benefits observed in the oldest age groups and in patients with complete treatment adherence (Paraskevis et al., 2023). However, real-world data have also highlighted important safety considerations. Mazzitelli et al., in a cohort of 909 patients treated with oral antivirals, found that adverse events occurred in 19.1% of N/R recipients—substantially higher than the rates reported in the pivotal EPIC-HR trial—with dysgeusia (12.4%) and diarrhea (3.0%) being the most frequently reported, and treatment discontinuation occurring in 4.8% of patients (Mazzitelli et al., 2023). These real-world effectiveness and safety data underscore the importance of individualized therapy and support the potential role of therapeutic drug monitoring (TDM) in optimizing N/R use, particularly in vulnerable populations such as older adults, patients with multiple comorbidities, and those receiving polypharmacy.

Despite the effectiveness of N/R in combating COVID-19, the inhibition of CYP3A metabolism by ritonavir can lead to a rapid increase in the concentration of drugs such as tacrolimus and cyclosporine, which are also metabolized by CYP3A4. This increase can result in adverse effects such as brain damage, epilepsy, kidney damage, and even death (Luo et al., 2016; Prikis and Cameron, 2022; Hashemian et al., 2023; Lemaitre et al., 2023). N/R is not recommended for patients with severe renal or hepatic impairment. Therefore, for patients taking N/R, especially those with special conditions, timely monitoring of drug concentration is crucial for effective treatment. Therapeutic drug monitoring (TDM) has become an important tool for optimizing the use of biopharmaceuticals. It is primarily used to monitor drugs with challenging target concentrations, significant pharmacokinetic variability, and adverse reactions (Bassetti et al., 2017; Bienvenu et al., 2021). Therefore, based on previous experience (Kang and Lee, 2009; Papamichael et al., 2022), TDM may be an optimized tool for drug therapy to manage drug-drug interaction (DDI), specifically for patients using potential related medications.

Given the critical role of adequate nirmatrelvir exposure in achieving therapeutic efficacy and the high inter-individual variability in plasma concentrations, we identified several factors that could potentially influence N/R blood levels, providing a foundation and reference for the clinical use of N/R. The primary objective of this study was to identify key baseline clinical and laboratory factors that determine nirmatrelvir exposure levels in high-risk outpatients with COVID-19, and to develop a machine learning model to predict individual drug exposure based on these readily available pretreatment variables. This approach aims to inform future strategies for personalized dosing or therapeutic drug monitoring (TDM) to optimize treatment outcomes.

## 2 Patients and methods

### 2.1 Study design

We identified several factors that could potentially influence N/R blood levels, providing a foundation and reference for the clinical use of N/R. This retrospective multicenter study included all patients who received N/R treatment at Changxing People’s Hospital and Second Affiliated Hospital of Xi’an Jiaotong University from December 2022 to June 2023. Informed consent forms were signed by all patients. All patients were administered two tablets of nirmatrelvir and one tablet of ritonavir orally or nasally every 12 hours. Patients are advised to take nirmatrevir and ritonavir simultaneously. The maintenance dose was 300 mg administered for 5 days.

### 2.2 Patients enrollment

Inclusion criteria: age≥18 years; patients adhered to the prescribed medication regimen. Blood samples were collected from all patients on the third day after drug administration, 30 minutes prior to drug administration. At least one sample from each patient was tested for TDM. If patients discontinued medication for reasons other than the study, the time of re-administration was considered as the initial treatment. The primary disease under investigation was neocoronitis. Exclusion criteria: incomplete clinical data recording, failure to meet blood sample collection and testing requirements.

### 2.3 Sample size consideration

Given the observational nature of this study and the absence of preliminary data on factors influencing N/R concentrations, a formal sample size calculation was not performed *a priori*. However, we conducted a *post-hoc* power analysis based on the detected effect size for eGFR (the most important predictor) in the random forest model. With a significance level of α = 0.05 and a desired power of 0.80, the minimum sample size required to detect a moderate effect (f² = 0.15) in a multiple regression model with 8 predictors was estimated to be 108. Our final sample of 139 patients exceeds this threshold, providing adequate power for the primary predictive analyses. Nevertheless, we acknowledge that the sample is modest for machine learning applications, and external validation in larger cohorts is warranted.

### 2.4 Clinical data and definitions

At baseline, we collected and recorded the following information from patients: sex, age, department, diagnosis, smoking and alcohol abuse history, underlying diseases (hypertension), blood routine index, liver function indicators (alkaline phosphatase [ALP], glutamyl transpeptidase [GGT], aspartate transaminase [AST], and alanine transaminase [ALT]), renal function indicators (creatine kinase [CK], creatinine, and estimated glomerular filtration rate [eGFR]), clinical symptoms, and use of CYP inducers and inhibitors.

Prognostic outcomes were assessed at day 28 or at hospital discharge, whichever occurred first. Patients were classified into two groups:

Group 1 (favorable prognosis): Patients who achieved clinical improvement or cure, defined as resolution of COVID-19-related symptoms (total symptom score ≤ 2) and discharge from hospital without requiring supplemental oxygen.

Group 0 (unfavorable prognosis): Patients who experienced worsening of clinical status or death. Worsening was defined as any of the following: (i) progression from mild/moderate to severe/critical disease requiring intensive care unit (ICU) admission; (ii) initiation of invasive mechanical ventilation; (iii) development of new organ failure (e.g., acute kidney injury, acute respiratory distress syndrome); or (iv) death from any cause during the follow-up period.

### 2.5 Quantification of nirmatrelvir and ritonavir plasma concentrations

#### 2.5.1 Chromatographic and mass spectrometric conditions

A methodology for the analysis of N/R in the body has been established using UPLC-MS/MS (Fan et al., 2024). Specifically, we utilized an ACQUITY UPLC BEH C18 (2.1 mm×50 mm, 1.7 μm) column, with 100% acetonitrile as the A-phase and water containing 0.1% formic acid as the B-phase. To enhance sample separation and improve separation efficiency, we performed gradient elution on the samples. The gradient elution consisted of the following conditions: 0-1.2 min, 22% A; 1.2-1.9 min, 100% A; 1.9–2 min, 100% A; 2–3 min, 22% A. The elution was carried out at a flow rate of 0.3 mL/min, with a column temperature of 45°C and an injection volume of 2μL.

The electrospray ionization source (ESI)+ mode was utilized, with a capillary voltage of 3000 V, a desolvation temperature of 350°C, an N_2_ flow rate of 600 L/h, and a scanning mode of MRM. Positive and negative ions were alternated for scanning and analysis, and the parameters for mass spectrometry detection are provided in the table below (**Table 1**).

**Table 1:** Mass spectrometric parameters of analytes and internal standards.

Detected ions	Precursor ion	Product ion	Cone/V	Collision/V
Nirmatrelvir	500.20	319.10	10	20
Nirmatrelvir-D_9_	508.59	328.10	10	20
Ritonavir	721.30	426.10	20	20
^13^C,^2^H_3_- ritonavir	725.30	426.10	20	20

#### 2.5.2 Preparation of reserve solution and working solution

The Nirmatrelvir and ritonavir standards were accurately weighed. Nirmatrelvir and its internal standard, nirmatrelvir-D_9_, were dissolved in acetonitrile, while ritonavir and its internal standard, ^13^C, ^2^H_3_--ritonavir, were dissolved in acetonitrile: water (50:50, V/V). Nirmatrelvir and ritonavir were prepared as separate standard stock solutions with a concentration of 1.00 mg/mL each. Nirmatrelvir-D9 and ^13^C, ^2^H_3_-ritonavir were dissolved separately, mixed, and then diluted with acetonitrile: methanol (3:1, V/V) containing 0.1% formic acid. This resulted in a mixed internal standard working solution with concentrations of 1.00 μg/mL for nirmatrelvir- D_9_ and 0.50 μg/mL for ^13^C, ^2^H_3_-ritonavir.

Take the appropriate amount of nirmatrelvir and ritonavir standard stock solution. Dilute them step by step with acetonitrile: water (3:1, V/V) containing 0.1% formic acid to obtain a series of mixed standard working solutions. The concentrations of nirmatrelvir in the solutions are 300, 100, 50, 20, 10, 5, and 1 μg/mL, while the concentrations of ritonavir are 50, 40, 20, 10, 5, 1, and 0.5 μg/mL. Additionally, prepare low concentration quality control (QC) samples with nirmatrelvir at 5 μg/mL and ritonavir at 1 μg/mL, medium concentration QC samples with nirmatrelvir at 10 μg/mL and ritonavir at 10 μg/mL, and high concentration QC samples with nirmatrelvir at 80 μg/mL and ritonavir at 40 μg/mL.

#### 2.5.3 Preparation of standard curve and quality control

The standard curve and QC were prepared by adding 10 μL of a mixed standard working solution containing nirmatrelvir and ritonavir to 90 μL of blank human plasma. The standard curve for nirmatrelvir ranged from 30, 10, 5, 2, 1, 0.5, 0.1 μg/mL, while the standard curve for ritonavir ranged from 5, 4, 2, 1, 0.5, 0.1, 0.05 μg/mL. The low concentration levels were 0.5 μg/mL for nirmatrelvir and 0.1 μg/mL for ritonavir. The medium concentration levels were 1 μg/mL for nirmatrelvir and 1 μg/mL for ritonavir. The high concentration levels were 8 μg/mL for nirmatrelvir and 4 μg/mL for ritonavir. The lower limit of quantification was 0.1 μg/mL for nirmatrelvir and 0.05 μg/mL for ritonavir.

#### 2.5.4 Sample pretreatment

Transfer 100 μL of plasma sample into a tube, then add 300 μL of a mixed internal standard working solution. Mix the contents thoroughly and centrifuge at 12000 rpm/min for 5 minutes. Finally, take 2 μL of the supernatant for analysis.

### 2.6 Statistical analysis

#### 2.6.1 Data preprocessing and multicollinearity assessment

All statistical analyses were performed using R software (version 4.3.0). Continuous variables are presented as median (interquartile range, IQR) and categorical variables as frequencies (percentages). Pairwise Spearman correlation was computed for all candidate predictors. When two features exhibited an absolute correlation coefficient > 0.5 and *P* < 0.05, the clinically less relevant or less complete variable was removed. This step was performed to mitigate multicollinearity before feature selection.

#### 2.6.2 Logistic regression for prognosis association

To assess whether nirmatrelvir trough concentration alone could discriminate between patients with favorable (cured/improved) versus unfavorable (worsened/death) outcomes, a logistic regression model was fitted with concentration as the sole independent variable. Receiver operating characteristic (ROC) curve analysis was performed and the area under the curve (AUC) was computed (Obuchowski and Bullen, 2018). Because the outcome was imbalanced (114 favorable vs. 25 unfavorable), cost-sensitive learning was applied by weighting observations inversely to their class frequencies. In addition, an iterative sensitivity analysis was conducted: the sample with the highest nirmatrelvir concentration was removed, the model was refitted, and changes in AUC and threshold were monitored. This process was repeated until no further improvement was observed, ensuring robustness against extreme values.

### 2.7 Machine learning model development and validation

#### 2.7.1 Feature selection using random forest

The dataset was randomly split into training (n = 112) and validation (n = 27) sets at a 4:1 ratio using stratified sampling based on the distribution of the outcome and eGFR categories to maintain representativeness. A random forest regression model(18) (target: nirmatrelvir trough concentration) was built on the training set using the randomForest R package with the following settings: number of trees = 1000, number of variables tried at each split (mtry) = default (one-third of predictors), node size = 5. Variable importance was measured as the percentage increase in mean squared error (%IncMSE) when a feature was permuted. Features with positive %IncMSE were considered potentially influential and were retained for subsequent predictive modeling.

#### 2.7.2 XGBoost model development and hyperparameter optimization

An XGBoost regression model was trained on the same training set using the features selected by random forest. To prevent overfitting and optimize generalization, a grid search of hyperparameters was performed using five-fold cross-validation on the training data. The following parameters were tuned: learning rate (eta = 0.1, 0.15, 0.2, 0.25, 0.3), subsample ratio (subsample = 0.6, 0.8, 1.0), and maximum tree depth (max_depth = 4, 6, 8). The number of boosting rounds (nrounds) was set to 500 with early stopping if validation error did not improve for 50 consecutive rounds. The combination yielding the lowest cross-validated RMSE was selected. Learning curves (RMSE vs. boosting iterations) were plotted for different hyperparameter combinations to visualize the bias-variance trade-off.

#### 2.7.3 Model evaluation

The final XGBoost model was evaluated on the held-out validation set (n = 27). Predictive accuracy was quantified by mean absolute error (MAE), mean squared error (MSE), root mean squared error (RMSE), and the coefficient of determination (R²). Additionally, internal validity was assessed through five-fold cross-validation repeated five times; the average performance metrics across folds are reported. Feature importance in the XGBoost model was expressed by three metrics: Gain (average improvement in accuracy when a feature is used in a split), Cover (average coverage of splits using that feature), and Frequency (relative number of times a feature is used).

All code used for data preprocessing, feature selection, model training, and evaluation is available from the corresponding author upon reasonable request. Key random seeds were set to ensure reproducibility.

## 3 Results

### 3.1 Study population and baseline characteristics

A total of 139 high-risk outpatients with mild-to-moderate COVID-19 treated with nirmatrelvir/ritonavir were included. The cohort had a median age of 74 years [interquartile range (IQR): 62–83], with 77 (55.4%) male and 62 (44.6%) female patients. Baseline characteristics, including comorbidities and laboratory parameters, are summarized in **Table 2**. Key variables relevant to drug metabolism—such as estimated glomerular filtration rate (eGFR, median 78 mL/min/1.73m² [65–92]), alanine aminotransferase (ALT, median 28 U/L [20–40]), and procalcitonin (PCT, median 0.12 ng/mL [0.06–0.25])—exhibited considerable variability across the cohort. All continuous variables were non-normally distributed (Shapiro-Wilk test p < 0.05) and are therefore reported as median [IQR].

**Table 2:** Baseline characteristics of the included patients (N = 139).

Variables	Level	COVID-19		*P*-value
		Group 1 (114), n (%)/ median (IQR)	Group 0 (25), n (%)/ median (IQR)	
Sex	Male	60 (52.63%)	17 (68%)	0.164
Age	year	75 (64 - 83)	81 (73 - 89)	0.009
History of smoking	Yes	12(10.53%)	2 (8%)	0.989
	No	102 (89.47%)	23 (92%)
History of alcoholism	Yes	6 (5.26%)	1 (4%)	1
	No	108 (94.74)	24 (96%)
Underlying disease of hypertension	Yes	49 (42.98%)	16 (64%)	0.092
	No	65 (57.02)	9 (36%)
Department	ICU	5 (4.39%)	13 (52%)	**/**
	Respiratory and Critical Care Medicine	53(46.49%)	7 (28%)	**/**
	Cardiovascular	4 (3.51%)	1 (4%)	**/**
	Oncology and hematology	6 (5.26%)	0 (0%)	**/**
	Neurology	5 (4.39%)	0 (0%)	**/**
	Infectious diseases	11 (9.65%)	0 (0%)	**/**
	Nephrology	3 (2.63%)	2 (8%)	**/**
	Gastroenterology	1 (0.88)	1 (4%)	**/**
	Urological	1 (0.88)	0 (0%)	**/**
	Gynecology and obstetrics	1 (0.88)	0 (0%)	**/**
	Orthopedic surgery	1 (0.88)	0 (0%)	**/**
	Emergency medicine	3 (2.63%)	0 (0%)	**/**
	Endocrinology	2 (1.75%)	0 (0%)	**/**
	General medicine	13 (11.4%)	1 (4%)	**/**
	Rehabilitation medicine	2 (1.75%)	1 (4%)	**/**
	Gerontology	2 (1.75%)	0 (0%)	**/**
ALT	U/L	23.56 (18.89 - 32.25)	25.46 (9.2 - 36.18)	0.884
AST	U/L	23.96 (16.74 - 34.12)	30.73 (16.9 - 44.45)	0.274
eGFR	mL/minute	55.92 (43.62 - 69.5)	41.75 (20.33 - 55.92)	<0.001
CRP	mg/L	31.8 (7.23 - 82.08)	113.1 (33.7 - 140.8)	0.001
PCT	ng/mL	0.06 (0.03 - 0.08)	0.65 (0.06 - 2.29)	<0.001
ALP	U/L	65.9 (54 - 79)	69 (54 - 85)	0.641
GGT	U/L	31.30 (19.56 - 49.98)	40.01 (26.93 - 55)	0.329
CK	U/L	2.91 (2.1 - 3.5)	2.94 (2.59- 3.93)	0.348
Cr	μmol/L	81.1 (64.78 - 101.58)	100.3 (82.2 - 163.3)	0.003
WBC	×10^9^/L	5.56 (4.2 - 8.3)	7.5 (5 - 11.9)	0.037
NE	×10^9^/L	4.25 (3 - 6.9)	6.2 (4.4 - 10.2)	0.013
Lymph	×10^9^/L	0.69 (0.5 - 1.28)	0.6 (0.3 - 0.8)	0.004

Cr, Creatinine; CRP, C-reaction protein; WBC, White blood cell; NE, Neutrophil; lymph, Lymphocyte; PCT, Procalcitonin.

Group 1: Patients with favorable prognosis (clinical improvement or cure). Group 0: Patients with unfavorable prognosis (clinical worsening or death). Worsening includes ICU admission, mechanical ventilation, new organ failure, or death.

### 3.2 Changes in clinical symptom score

**Figure 1:**
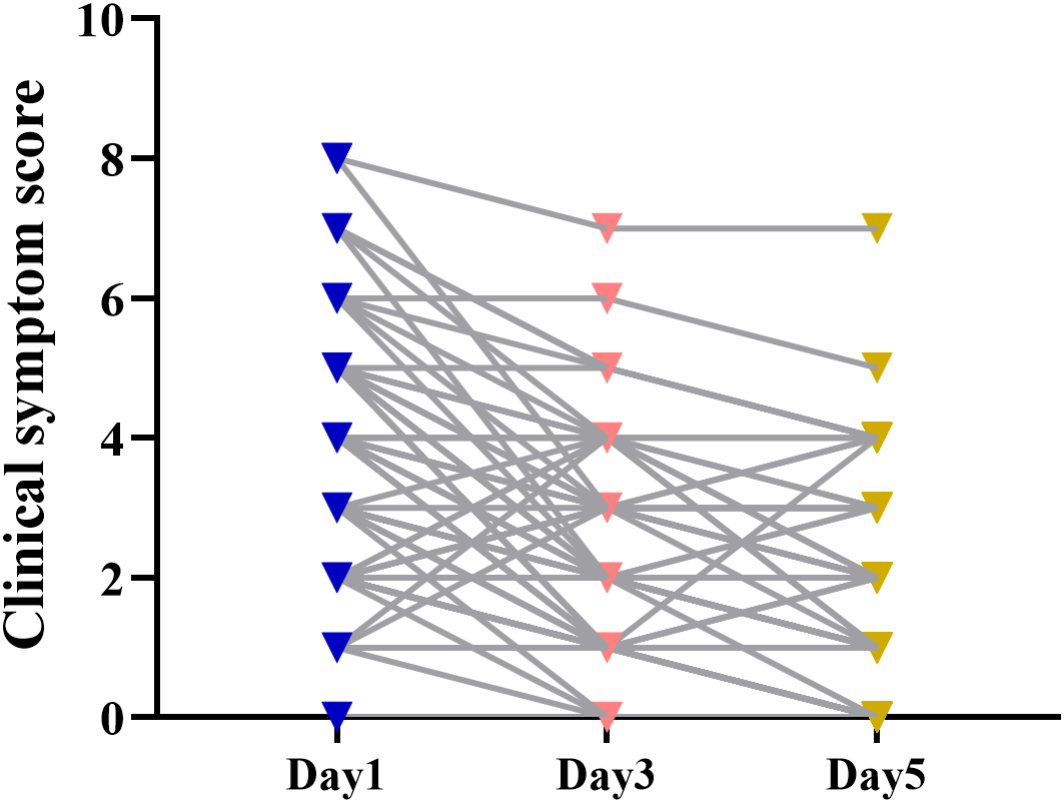
Changes in clinical symptom score. Blue is the symptom score of all patients on day 1, pink is the symptom score of all patients on day 3, and yellow is the symptom score of all patients on day 5.

We assessed the severity of common symptoms in COVID-19 patients, including fever, cough, nasal congestion, headache, muscle ache, nausea, chills, vomiting, and diarrhea, using a quantitative scoring system (0-none, 1-mild, 2-moderate, 3-severe). As depicted in **Figure 1**, the clinical symptom scores of nearly all patients showed a gradual decrease over the course of N/R administration. The aforementioned symptoms exhibited a gradual decrease in patients who received continuous N/R administration. These findings suggest that N/R exhibits efficacy in the treatment of SARS-CoV-19, and the timing of administration may influence its effectiveness.

### 3.3 Feature correlation analysis

**Figure 2:**
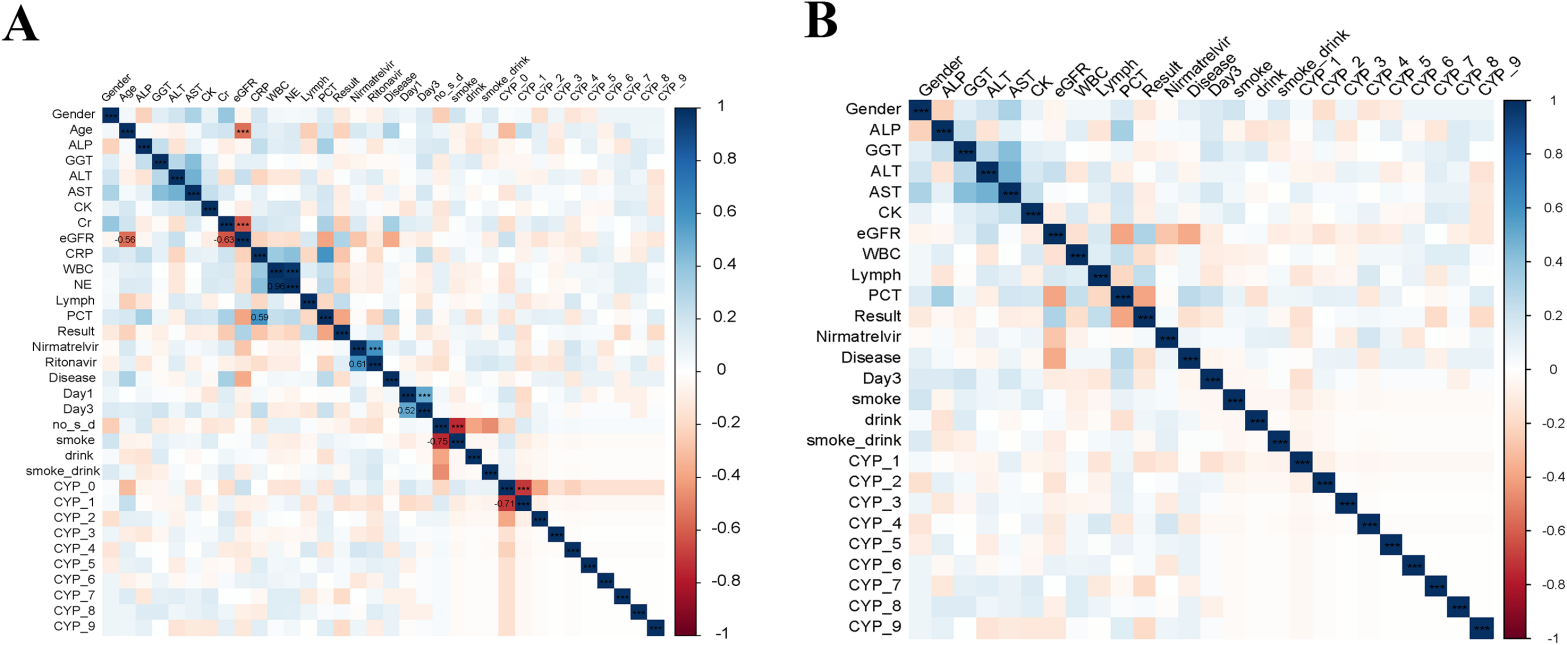
Correlation matrix between features. **(A)** Spearman correlation analysis between all features. **(B)** Spearman correlation analysis after excluding significantly correlated features. Disease represents hypertensive. Blue color indicates positive correlation, red color indicates negative correlation, and dark color indicates stronger correlation. ***p < 0.001.

Before performing feature selection, we conducted a correlation analysis on all variables to identify and eliminate highly correlated features. Highly correlated features were defined as those with an absolute correlation coefficient greater than 0.5 and a P-value less than 0.05. **Figure 2A** shows the Spearman correlation heatmap for all features, and **Figure 2B** displays the heatmap after removing features with correlations beyond the defined threshold. Stronger correlations are represented by darker shades. We observed a strong correlation between the concentrations of nirmatrelvir and ritonavir (Correlation: 0.6063156, *P*<0.001). Therefore, we decided to analyze only the concentrations of nirmatrelvir in the subsequent analyses.

### 3.4 Nirmatrelvir blood concentration predicts the prognosis

**Figure 3:**
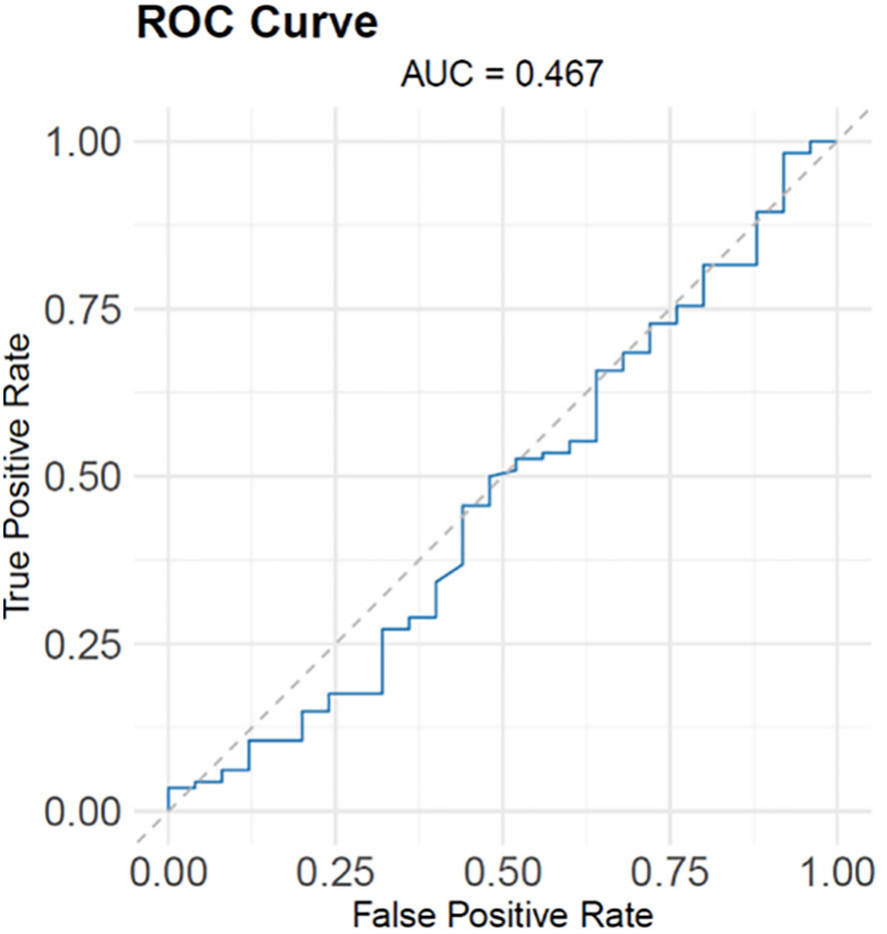
ROC analysis of nirmatrelvir blood concentrations for predicting prognosis.

We evaluated the predictive value of Nirmatrelvir blood concentration for COVID-19 patient outcomes using the receiver operating characteristic (ROC) curve. The area under the ROC curve (AUC) was 0.467, indicating that the model’s predictive ability is similar to random guessing. This finding suggests that Nirmatrelvir blood concentrations may not have a significant correlation with prognostic outcomes, indicating limited usefulness in predicting patient prognosis (**Figure 3**).

### 3.5 Feature selection from random forest model

**Figure 4:**
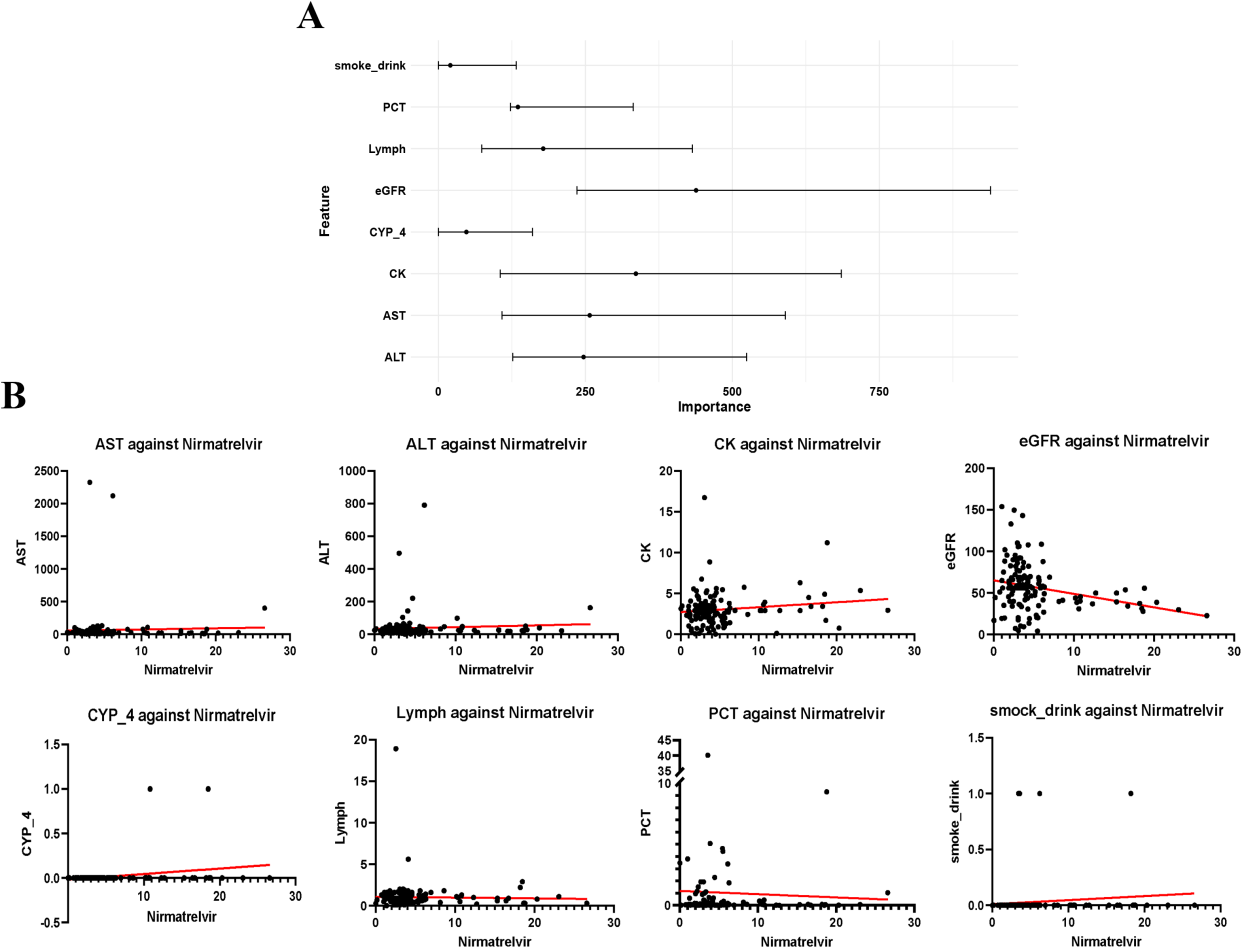
Feature screening results. **(A)** Feature Importance from Random Forest Model. **(B)**. Scatterplot showing linear relationship between characterization and blood drug concentration.

The Random Forest model was used to identify the features that had the greatest impact on our analysis. This model ranked the features based on their importance. The most important feature identified was the eGFR, which is an indicator of renal function. It was followed by creatine kinase, AST, ALT, Lymph, PCT, the use of the cytochrome P450 enzyme inhibitor omeprazole (CYP-4), and the patient’s smoking and drinking history. These results suggest that factors such as renal and liver function, lymphocyte and platelet counts, and lifestyle choices like smoking and alcohol consumption may significantly affect the metabolism and elimination of N/R, as shown in **Figure 4A**.

Further analysis was performed to investigate the relationship between these features and blood drug concentration. Scatter plots were created to visually represent the distribution of each feature’s value in relation to blood drug concentration. Linear regression was then applied to these distributions, and the resulting regression lines were used to illustrate the linear relationships between each feature and blood drug concentration, as depicted in **Figure 4B**.

### 3.6 Learning curve analysis for hyperparameter optimization

**Figure 5:**
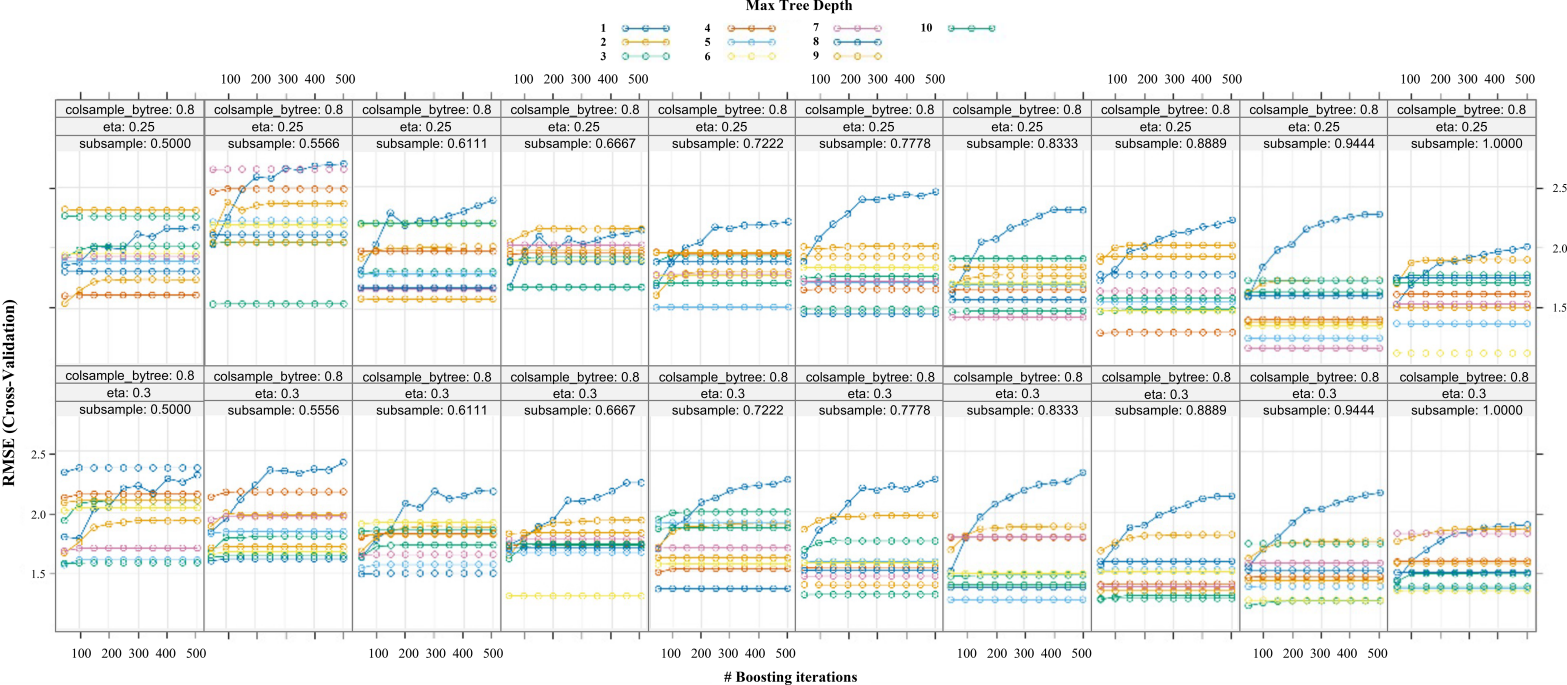
Learning curves for XGBoost model hyperparameter optimization. This figure displays the RMSE on the cross-validation set over 500 boosting iterations for various combinations of eta (learning rate) and subsample (sample random sampling rate) values. Each line corresponds to a different max tree depth, reflecting changes in model performance with increasing complexity.

After selecting important features using the random forest model, we trained the XGBoost model with these features to determine the optimal hyperparameters for the model’s generalization performance. We analyzed the learning curves to observe how the model’s error, measured by RMSE, changed with increasing boosting iterations under different settings of eta (learning rate) and subsample (random sampling rate of samples), as shown in **Figure 5**.

The results shown in **Figure 5** indicate that the model, with an eta of 0.25 and a subsample of 1, consistently achieved a low and stable RMSE. This suggests that the model had an appropriate learning rate and effectively utilized the dataset. Additionally, a max tree depth of 6 was found to be effective in maintaining the model’s performance without overfitting, even as the number of iterations increased.

### 3.7 XGBoost model

After training the XGBoost model with the optimally selected hyperparameters, we assessed the importance of each feature in the model. The model output includes three metrics of feature importance: Gain, Cover, and Frequency. ‘Gain’ represents the average gain of splits that utilize the feature, ‘Cover’ corresponds to the average coverage of splits that utilize the feature, and ‘Frequency’ indicates the proportion of times a feature is used in splits across all trees.

**Figure 6:**
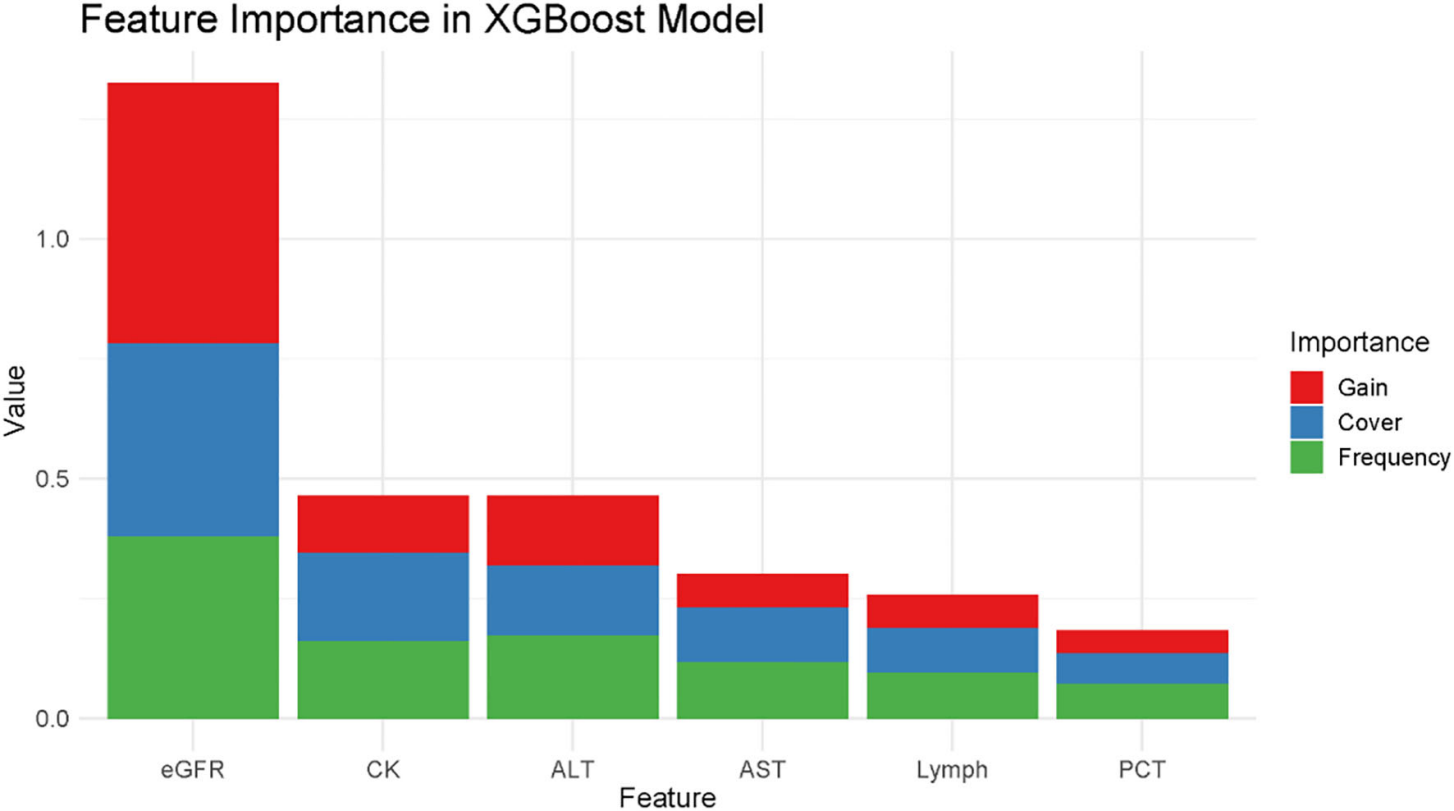
Feature importance in the trained XGBoost model. The stacked bar chart displays the relative importance of each feature according to three metrics: Gain (red), Cover (blue), and Frequency (green). The total height of each bar represents the cumulative importance of each feature.

The results, depicted in **Figure 6**, emphasize that eGFR has the highest gain among the features, implying its significant influence on model predictions. Additionally, liver enzymes ALT and AST, CK, Lymph, and PCT exhibit notable importance, signifying their relevance in the model’s decision-making process. Analyzing these metrics reveals the biological factors that are most predictive of blood drug concentrations based on the trained model (**Figure 6**).

We trained the XGBoost model with the selected features and hyperparameters, and then evaluated its predictive performance using the validation set. The model’s predictions were assessed using various statistical metrics to measure prediction error and goodness of fit. The mean squared error (MSE) was 1.328, indicating the average squared difference between the observed actual outcomes and the model’s predictions. The root mean squared error (RMSE), which measures error in the same units as the response variable, was 1.152. The mean absolute error (MAE), representing the average absolute difference between observed and predicted values, was 0.717. Additionally, the model achieved an R-squared (R²) value of 0.779, suggesting that approximately 77.9% of the variability in the response variable was explained by the model. As shown in **Table 3**, these metrics collectively demonstrate a strong predictive performance, with the model accurately forecasting blood drug concentrations.

**Table 3:** Predictive performance metrics of the XGBoost model on the validation set.

Indicator	Value
MSE	1.328
RMSE	1.152
MAE	0.717
R-squared	0.779

## 4 Discussion

In this multicenter study, we identified six routinely available clinical variables — eGFR, CK, AST, ALT, lymphocyte count, and PCT — as significant predictors of nirmatrelvir trough concentration using a combination of random forest and XGBoost models. These findings extend previous work by providing a multivariable predictive tool rather than relying on single biomarkers (Toth et al., 2019; Hammond et al., 2022; Park et al., 2023; Yip et al., 2023). TDM assists clinicians in tailoring dosage adjustments for patients’ medication, maximizing drug efficacy, and minimizing adverse drug reactions by considering individual differences in pharmacokinetics (Luong et al., 2016; Fang et al., 2024). While TDM has been used for clinical monitoring of various drugs, no study has investigated the potential factors influencing N/R blood concentration in different patients using TDM. Therefore, it is promising and highly meaningful for TDM to guide individualized dosing in the clinical study of N/R.

In our baseline data, we observed that patients with a prognosis of worsening or death were significantly older compared to those with an improved or cured prognosis, which aligns with previous findings. Age is a widely recognized risk factor for COVID-19 (Gao et al., 2021; Zhang et al., 2023). Older adults exhibit weakened immune systems, making them more vulnerable to viral infections and prone to immune system overactivation caused by chronic inflammation, potentially resulting in pneumonia. Furthermore, compromised organ function and pre-existing chronic conditions in older adults play crucial roles in pneumonia development. The slowing down of the body’s metabolism with increasing age suggests that elderly individuals need regular monitoring of blood levels and adjustment of the therapeutic regimen when using N/R. Additionally, a wide range of laboratory biochemical indicators are considered the main risk factors for the severity and mortality of COVID-19. Our study found significant differences in eGFR, CRP, PCT, Cr, WBC, NE, and Lymph in both groups, which is consistent with the findings of a previous study (Gao et al., 2021). Differences in biochemical parameters may be associated with the prognosis of patients. On one hand, they can alter the metabolism and absorption of drugs, and on the other hand, they can influence the response of the immune system. During inflammation, the immune system stimulates a rapid increase in WBC and NE to combat pathogenic bacteria. Additionally, Lymph is massively translocated to the site of inflammation to recognize and destroy virus-infected cells. Elevated levels of CRP and PCT, which are markers of acute inflammation, indicated the presence of severe inflammatory infections in our patients in the group with worsening prognosis or death. While a small percentage of patients on N/R still failed to recover from COVID-19, the majority of patients had a favorable prognosis, indicating the beneficial effects of N/R against COVID-19.

In the study conducted by Cao et al (Cao et al., 2023), all patients in the nirmatrelvir-ritonavir group achieved sustained clinical recovery. Clinical recovery was defined as the remission of all target symptoms related to COVID-19, with a total score of 0 or 1 for each symptom. Consistent with their findings, our study involved patients who received a maintenance dose of N/R for 3 days. During this period, there was a gradual decrease in clinical symptom scores. On the fifth day, the majority of patients (111 cases) had a total score of less than 2, indicating significant relief from symptoms. We can hypothesize that the extended duration of N/R administration allowed nirmatrelvir to maintain high drug concentrations and sustained efficacy in patients. Despite the initial indication of a relationship between nirmatrelvir and patient outcomes, the logistic regression modeling analysis unexpectedly showed weak correlation between prognostic outcomes and nirmatrelvir blood concentrations. This could be attributed to the older age and presence of severe underlying diseases, including cardiovascular disease (including hypertension), diabetes, and leukemia, among the patients. The poorer prognostic outcome of the patients was likely a result of the underlying disease, causing organ dysfunction, failure, or excessive immunocompromise, unrelated to SARS-CoV-2 infection.

N/R blocks the activity of the SARS-COV-2-3Cl protease, which is necessary for coronavirus replication, to exert its antiviral effect (Eng et al., 2022; Reina and Iglesias, 2022; Wen et al., 2022). After excluding highly correlated individual features, Spearman correlation analyses revealed a high correlation between nirmatrelvir and ritonavir concentrations, indicating that low-dose ritonavir had an effect. Nimatrevir alone may be eliminated by the body due to lack of recognition. The combination of nimaltegravir and ritonavir slows down the metabolism and breakdown of PF-07321332, thereby prolonging its high activity and exerting antiviral effects. The random forest model screened eight important features that affect blood drug concentrations by comparing the training cohort (n = 112) with the validation cohort (n = 27). Interestingly, the most important feature was eGFR, followed by Creatine Kinase, PCT, AST, ALT, Lymph, CYP4, and smoke-drink. The dominant role of eGFR is physiologically plausible. Nirmatrelvir is primarily excreted renally, and reduced kidney function predictably leads to higher drug accumulation. Our results align with current prescribing recommendations that dose adjustment is required when eGFR < 60 mL/min. Previous studies reported significantly higher Creatine kinase concentrations in deceased patients compared to recovered patients (Chen et al., 2020). Creatine kinase is a risk factor for patient severity and mortality (Malik et al., 2021; Izcovich et al., 2022). In this study, we discovered for the first time that both Creatine kinase and PCT have significant effects on N/R blood concentrations, which is a meaningful finding. During inflammation, cells are destroyed and release creatine kinase into the bloodstream, leading to elevated concentrations. Elevated levels of creatine kinase in the blood indicate substantial damage to existing cells, which affects the absorption of N/R. PCT, which is normally expressed at low levels, is released in large quantities into the bloodstream during infection and is considered an important indicator for assessing the severity of severe infections. In our study, we found that PCT had a significant effect on N/R concentration. The PCT results in the good prognosis group showed mild infection in patients (0.5–2 ng/mL), while in the poor prognosis outcome group, PCT reached 3.12 ± 8.01 ng/mL, indicating severe infection in patients. N/R inhibits the replication of the SARS-CoV-2 virus, but it does not affect established viral loads. In severe cases, where the viral load is high, the inhibition of viral replication alone is limited. Nirmatrelvir is metabolized and eliminated from the body when it fails to exert its antiviral effect. Therefore, the range of PCT significantly affects the concentration of nirmatrelvir and ritonavir. However, we emphasize that the observed associations are correlational, not causal. Elevated CK and PCT may simply identify sicker patients who also have intrinsically lower N/R clearance due to other unmeasured factors. The liver plays a crucial role in drug metabolism, and AST and ALT are commonly used to assess liver damage. Elevated AST and ALT levels indicate impaired liver function, leading to reduced metabolism of N/R and affecting its efficacy. Lymphopenia, a hallmark of severe COVID−19, was inversely related to N/R exposure, possibly as a proxy for overall disease severity rather than a direct pharmacokinetic effect. Additionally, omeprazole (CYP-4) may also play a significant role in influencing the concentration of N/R. Omeprazole down-regulates immunoglobulin E (IgE) receptor sites on plasma cell-like dendritic cells, leading to increased production of interferon alpha (INF-alpha) and enhanced antiviral immunity. We hypothesize that this could be attributed to the alteration of immune status by omeprazole, which may affect the cellular metabolism of N/R through immunokines or immune cells. The primary function of lymph is to maintain immune system homeostasis and to identify and eliminate abnormal cells.

This study represents the first application of the XGBoost modeling algorithm to identify the features that influence the concentration of N/R in the blood. Through careful selection of hyperparameters, we optimized the model’s performance in validation, striking a balance between complexity and generalization to mitigate the risk of overfitting. Learning curves played a crucial role in this process, allowing us to iteratively fine-tune the model until we achieved the optimal configuration. The XGBoost model identified six features, namely eGFR, CK, AST, ALT, Lymph, and PCT, as significant predictors of N/R concentration levels in COVID-19 patients. These features were consistent with those identified by the Random Forest model, thereby strengthening the validity of our feature selection process. The identification of these key features highlights the potential physiological and metabolic factors that may impact the response of patients to N/R. Integrating these findings into clinical practice could improve the therapeutic monitoring of N/R, potentially leading to more personalized and effective treatment strategies for COVID-19 patients. From a clinical perspective, the ability to predict N/R exposure using routinely measured laboratory tests could enable preemptive dose adjustments in high-risk patients (e.g., those with severely reduced eGFR or marked inflammation). This would align N/R therapy with the broader trend toward precision dosing in infectious diseases.

It is important to acknowledge the limitations of the current study. First, the sample size (n = 139) is modest for ML studies; although we used cross−validation and early stopping, external validation in a larger, multi−institutional cohort is essential before clinical application. Second, we measured only a single trough concentration on day 3, which may not capture intra−individual pharmacokinetic variability. Full pharmacokinetic profiling with multiple sampling points would provide more precise exposure metrics. Third, unmeasured confounders (e.g., genetic polymorphisms, body weight, concomitant use of other interacting drugs not recorded) could influence N/R concentrations and were not considered. Fourth, the predictive model was developed for nirmatrelvir only, because ritonavir concentrations were highly correlated and added little independent information. Whether a model for ritonavir would offer additional clinical value is unclear. Finally, we did not perform external validation; therefore, our model should currently be regarded as a proof−of−concept requiring further testing.

## 5 Conclusion

We identified a set of variables through therapeutic drug monitoring (TDM) to influenced N/R exposure levels and developed a new machine-learning model to identify them. These variables offer valuable predictive information about patients’ N/R exposure levels and prognosis. Furthermore, we provide approximate ranges of nirmatrelvir and ritonavir concentrations, which can guide the establishment of standardized ranges for steady-state concentrations. Clinicians can refer to our findings to customize management strategies for patients, while researchers can utilize our findings to develop multivariate prognostic models for enhancing patient prognosis.

## Data Availability

The original contributions presented in the study are included in the article/supplementary material. Further inquiries can be directed to the corresponding authors.
